# Role of CyPA in cardiac hypertrophy and remodeling

**DOI:** 10.1042/BSR20193190

**Published:** 2019-12-23

**Authors:** Mengfei Cao, Wei Yuan, Meiling Peng, Ziqi Mao, Qianru Zhao, Xia Sun, Jinchuan Yan

**Affiliations:** Department of Cardiology, Affiliated Hospital of Jiangsu University, Zhenjiang, Jiangsu 212000, China

**Keywords:** Cardiac hypertrophy and remodeling, Cyclophilin A, Inflammation, Matrix metalloproteinases, Oxidative stress, Vascular injury

## Abstract

Pathological cardiac hypertrophy is a complex process and eventually develops into heart failure, in which the heart responds to various intrinsic or external stress, involving increased interstitial fibrosis, cell death and cardiac dysfunction. Studies have shown that oxidative stress is an important mechanism for this maladaptation. Cyclophilin A (CyPA) is a member of the cyclophilin (CyPs) family. Many cells secrete CyPA to the outside of the cells in response to oxidative stress. CyPA from blood vessels and the heart itself participate in a variety of signaling pathways to regulate the production of reactive oxygen species (ROS) and mediate inflammation, promote cardiomyocyte hypertrophy and proliferation of cardiac fibroblasts, stimulate endothelial injury and vascular smooth muscle hyperplasia, and promote the dissolution of extracellular matrix (ECM) by activating matrix metalloproteinases (MMPs). The events triggered by CyPA cause a decline of diastolic and systolic function and finally lead to the occurrence of heart failure. This article aims to introduce the role and mechanism of CyPA in cardiac hypertrophy and remodeling, and highlights its potential role as a disease biomarker and therapeutic target.

## Introduction

Cardiac hypertrophy and remodeling occur to compensate for internal or external stress before the onset of heart failure. Although this change is initially an adaptive response to maintain cardiac function, sustained hypertrophic stimulation becomes maladaptive, and eventually develops into heart failure [1]. Cardiomyocyte hypertrophy, apoptosis, and excessive activation of cardiac fibroblasts play an important role in this process [2]. Under pressure and volume overload, as well as a variety of growth factors and hormone stimulation, pathophysiological changes such as hypertrophy and apoptosis will happen in cardiomyocytes as these are permanent cells that do not proliferate [3]. Myocardial fibroblasts are the most abundant cells in the heart [4]. Under certain stimuli, myocardial fibroblasts proliferate, undergo phenotypic conversion, and secrete large amounts of extracellular matrix (ECM). This then induces myocardial sclerosis and ventricular remodeling, which affect ventricular compliance. The changes above in cardiomyocytes and fibroblasts eventually lead to a decline in diastolic and systolic function and heart failure [5].

It is traditionally believed that the progression of pathological cardiac hypertrophy is irreversible; once it develops into severe heart failure, there are no other effective treatments besides heart transplantation [6]. It has been confirmed that reactive oxygen species (ROS) production and further activation of the inflammatory response are important mechanisms for cardiac hypertrophy and remodeling that lead to heart failure [7–14]. Excessive ROS generation triggers oxidative damage to nucleic acids, proteins, and biofilms, leading to cell dysfunction and apoptosis. However, clinical randomized trials using antioxidants such as vitamin E and β-carotene did not significantly reduce cardiovascular events [15], suggesting that the lack of specific antioxidative therapy is meaningless.

Cyclophilin A (CyPA) is a highly conserved and ubiquitous protein that was first discovered as a receptor for cyclosporin A (CsA) in cells [16]. Initially, CyPA was only detected inside cells. More in-depth studies of CyPA have determined that CyPA can be secreted outside of cells in response to ROS in various cells [17–19], and the amount of secretion increases as the degree of oxidative stress increases. This leads to vascular wall damage, proliferation and migration of cardiac fibroblasts, as well as the release of cytokines and the production of more ROS, which then induces cardiomyocyte hypertrophy and apoptosis through paracrine mechanisms; this is closely related to the occurrence and development of pathological cardiac hypertrophy [20–22].

## Structural features and functions of CyPA

**Figure 1:**
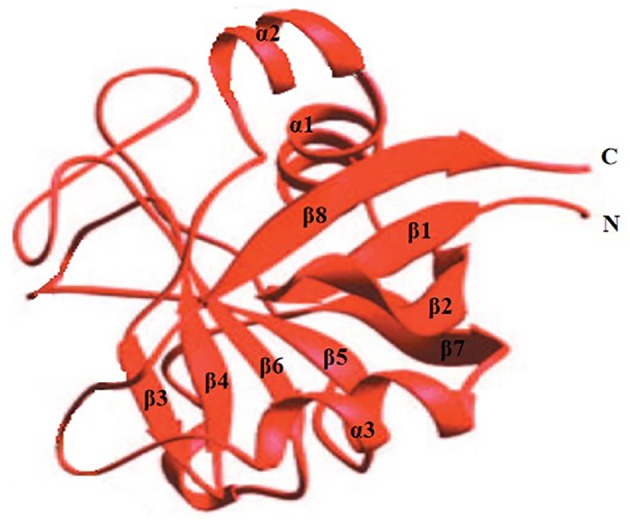
Schematic drawing of the structure of CyPs The α-helices are represented by a spiral designated as α, while β-strands are represented by an arrow marked as β. N and C denote the N and C termini of the molecule. Eight antiparallel strands (β1−β8) form a β-barrel and the two α-helices (α2 and α3) are on the top and bottom.

Cyclophilins (CyPs) belong to a group of a protein family that has peptidyl-prolyl cis–trans isomerase (PPIase) activity and is widely distributed in eukaryotes and prokaryotes [23]. CyPA was first purified from bovine thymocytes and was identified as the major cellular target for the immunosuppressive drug CsA [24]. The overall shape of CyPs is a flatted cylinder with three α-helices, eight β-strands, and six β-turns. Eight antiparallel β-strands constitute the surface, and two α-helices (α2 and α3) lie on the top and on the bottom of the cylinder, respectively (Figure 1). Seven aromatic residues (Phe8, Phe22, Phe36, Phe53, Phe112, Phe129, and Try48) and other hydrophobic residues form a hydrophobic core of the molecule within the cylinder [25]. A loop from Lys118 to His126 and four β strands (β3−β6) constitute a pocket structure, which is the binding site for CsA. Almost all the members of CyPs can bind to CsA [26,27].

CyPA has several functions including PPIase activity, protein folding, protein assembly and protein trafficking in cells (such as nuclear translocation of extracellular signal-regulated kinase (ERK1/2) and apoptosis-inducing factor (AIF)) [28], and involves many biological functions including immune regulation [29], cell growth [30], tumorigenesis [31], and cardiovascular diseases (for example, CyPA can promote the development of atherosclerosis, aortic aneurysm and cardiac hypertrophy) [32]. Subsequently, an increasing number of studies have shown that cardiomyocytes, cardiac fibroblasts, vascular smooth muscle cells (VSMCs), endothelial cells (ECs), and monocytes/macrophages can secrete CyPA to the outside of cells under the action of ROS. In VSMCs, CyPA is secreted through a process of vesicle formation, vesicle transport, and fusion at the plasma membrane [17]. Extracellular CyPA has not only similar cellular functions as intracellular CypA, such as proliferation and inflammation, but also unique characteristics, such as apoptosis, migration, matrix degradation, and generation of ROS. It must be noted that the ability of extracellular CyPA to produce ROS is likely to depend on its receptor, at least from the current findings [32].

## Receptor for CyPA in cardiovascular diseases

Yurchenko et al. first identified extracellular matrix metalloproteinase inducer (EMMPRIN, also known as CD147), which is encoded by basigin (Bsg), as a receptor for extracellular CyPA [33]. CyPA-induced chemotaxis and signaling pathways are achieved in two ways: one is through its PPIase activity and the other is through binding to EMMPRIN [34]. The mature form of EMMPRIN is a highly glycosylated transmembrane protein, which contains a short cytoplasmic domain, a transmembrane domain, and an extracellular domain [35]. EMMPRIN is highly expressed in many types of cells, including inflammatory cells and tumor cells. It is considered a matrix metalloproteinases (MMPs) inducing factor on the surface of tumor cells, which is involved in tumor invasion and metastasis [36]. In addition to the expression on tumor cells, EMMPRIN is also widely expressed on cardiovascular-related cells [37]. CyPA itself does not up-regulate the expression of EMMPRIN on the cell membrane, but after silencing EMMPRIN, the ERK1/2, nuclear factor κappa B (NK-κB) and Protein Kinase B (Akt) signaling pathways activated by CyPA are significantly attenuated [38,39]. These signaling pathways are involved in the development of pathological cardiac hypertrophy, including hypertrophic gene expression [40], fibrosis-related cell activation, migration and proliferation [41], regulation of the expression of various pro-inflammatory factors [42], and activation of MMPs [43]. Clinical studies have found that serum levels of EMMPRIN were significantly increased in patients with heart failure and were closely related to long-term survival [44]. After performing transverse aortic constriction (TAC) in Bsg^−/−^ (knockout) mice and in wild-type (WT) mice, Bsg^−/−^ mice showed reduced oxidative stress and MMPs activity in the left ventricle, less cardiac hypertrophy and cardiac interstitial fibrosis, as well as significantly improved long-term survival. Angiotensin II (AngII) or mechanical stretch induced cardiac fibroblasts express EMMPRIN. Up-regulation of EMMPRIN stimulated proliferation of cardiac fibroblasts by activating Akt and mitogen-activated protein kinases (MAPK) signaling pathways [45]. These data suggest that EMMPRIN plays a crucial role in the pathogenesis of cardiac hypertrophy, fibrosis, and heart failure, and may be a new biomarker and therapeutic target for pathological cardiac hypertrophy.

## CyPA in cardiac hypertrophy and remodeling

**Figure 2:**
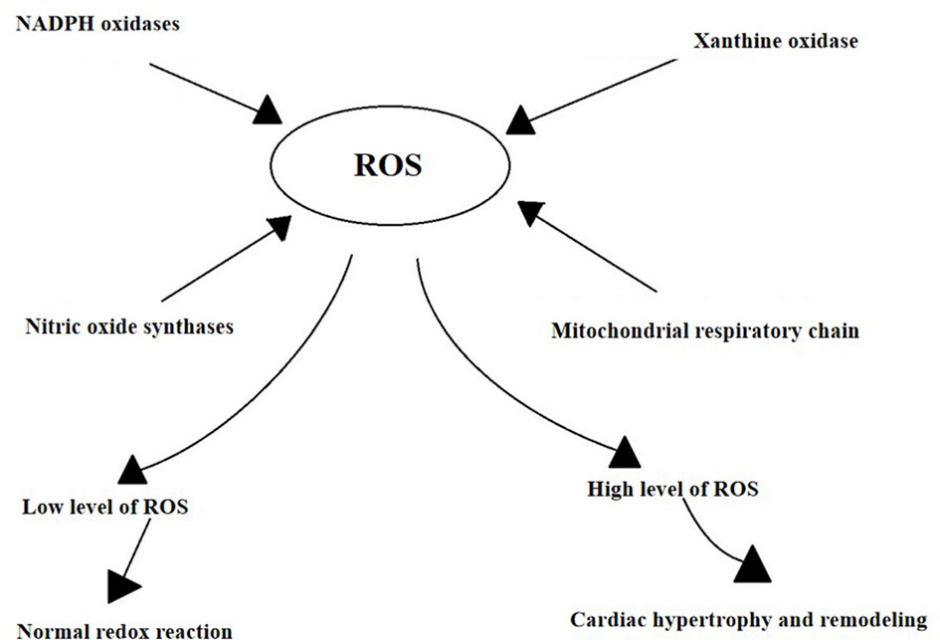
The possible mechanism of ROS production Low level of ROS is thought to play a role in controlling the normal redox reaction of physiological signaling pathways. High level plays a role in cardiac hypertrophy, apoptosis, ventricular remodeling, systolic, and diastolic dysfunction.

Pathological cardiac hypertrophy is often secondary to a variety of heart diseases, including hypertension, valvular heart disease, cardiomyopathy, and myocardial infarction. It is generally believed that the excessive production of ROS is an important mechanism for cardiac hypertrophy and remodeling [7–14]. ROS are generated by several mechanisms (Figure 2), including nicotinamide–adenine dinucleotide phosphate (NADPH) oxidases, xanthine oxidase (XO), the mitochondrial respiratory chain, and nitric oxide synthases (NOS) [10,13,14]. ROS formation can be stimulated by mechanical forces, (such as stretch, pressure, and shear stress), environmental factors (such as hypoxia), secreted factors coupled with tyrosine kinase receptors (such as platelet-derived growth factor (PDGF)), and secreted factors coupled with G-protein-coupled receptors (such as AngII, α, and β adrenergic receptor (αAR and βAR)) [46–49]. The human body produces a certain amount of ROS to control the normal redox reaction of physiological signaling pathways. Excessive ROS can cause cell dysfunction, lipid peroxidation, protein oxidation inactivation and DNA mutagenesis, leading to irreversible cell damage or death [10,13,14].

**Figure 3:**
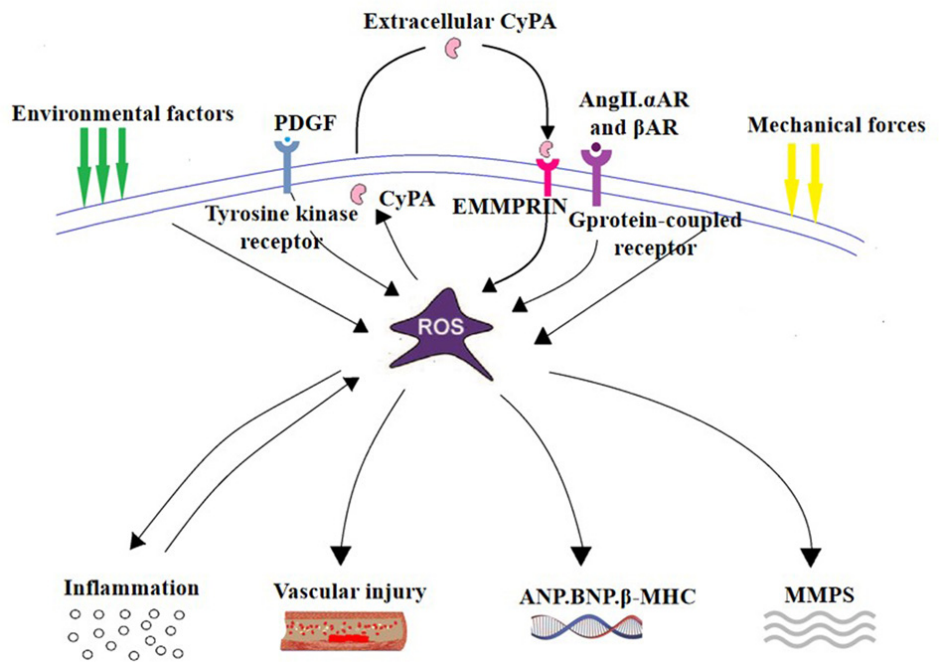
The main mechanism by which CyPA promotes cardiac hypertrophy and remodeling (1) CyPA is secreted in response to ROS, and then produces ROS to promote oxidative damage, mediates inflammatory response. (2) CyPA promotes the proliferation of cardiac fibroblasts and has a direct hypertrophic effect on cardiomyocytes. (3) CyPA stimulates vascular injury. (4) CyPA activates MMPs via EMMPRIN to promote ECM dissolution.

CyPA is a pathogenic protein that mediates oxidative stress-induced cardiovascular dysfunction, such as atherosclerosis [50], aortic dissection [15], and cardiac hypertrophy [51]. The production of CyPA and ROS is a vicious cycle. Under the condition of oxidative stress, excessive ROS stimulate the secretion of CyPA and activate related signaling pathways, further increasing the production of ROS [17–19]. *In vitro* and *in vivo* experiments have confirmed that CyPA can cause cardiac hypertrophy, and ventricular remodeling, and promote pathological cardiac hypertrophy [51–56]. The main mechanisms by which CyPA promotes cardiac hypertrophy and remodeling are characterized as follows: (1) CyPA produces ROS to promote oxidative damage, which mediates an inflammatory response. (2) CyPA directly promotes cardiomyocyte hypertrophy, myocardial fibroblast proliferation, and migration. (3) CyPA stimulates vascular endothelial injury and vascular smooth muscle hyperplasia. (4) CyPA activates MMPs via EMMPRIN to promote ECM dissolution (Figure 3).

### CyPA promotes oxidative stress and inflammatory response

Studies have shown that the concentration of CyPA and oxidative stress in plasma of patients with heart failure caused by various factors is significantly increased [52,55,56], suggesting that CyPA is involved as a pro-inflammatory and pro-oxidative factor in the process from cardiac hypertrophy and remodeling to heart failure. Animal experiments showed that after four weeks of Ang II infusion, the cardiac hypertrophy index increased significantly in both Ppia^−/−^ (CyPA knockout) and WT mice; however, there were no significant differences in the degree of cardiac hypertrophy between Ppia^−/-^ and WT mice [51]. Satoh hypothesized that the role of CyPA in cardiac hypertrophy requires a situation in which ROS production or inflammation is increased. The hearts of apolipoprotein E (ApoE^−/−^) mice have been shown to exhibit increased ROS production; moreover, CyPA is highly expressed in atherosclerotic plaques of ApoE^−/-^ mice, which has a pro-inflammatory effect on endothelial cells, leading to plaque progression [57]. When researchers treated ApoE^−/−^ mice and ApoE^−/−^Ppia^−/−^ mice with Ang II infusion, four weeks later they found that the cardiac weight, interventricular septum thickness, and cardiac myocyte size of ApoE^−/−^Ppia^−/−^ mice were significantly improved compared with ApoE^−/−^ mice. In addition, the cells that proliferate and migrate to the perivascular area are significantly reduced in ApoE^−/−^Ppia^−/−^ mice, as well as collagen content and fibrosis in the perivascular area. The production of ROS in the hearts and blood vessels of ApoE^−/−^Ppia^−/−^ mice was significantly reduced [51]. These data suggest that CyPA is secreted in the context of increased oxidative stress and inflammation, and acts as a determinant of ROS production to promote cardiac hypertrophy, remodeling, and vascular fibrosis in ApoE^−/−^ mice. Nigro et al. confirmed that CyPA is involved in the development of atherosclerosis by promoting oxidative stress and inflammation. Researchers simultaneously fed ApoE^−/−^ mice and ApoE^−/−^Ppia^−/−^ mice a high-fat diet. After 16 weeks, they found that the ApoE^−/−^Ppia^−/−^ mice developed significantly reduced atherosclerosis compared with ApoE^−/−^ mice, and CyPA deficiency was associated with decreased vascular cell adhesion molecule 1 (VCAM-1) expression, ROS, inflammation, ECs activation and apoptosis [57]. In a mouse model of Ang II-induced aortic dissection, CyPA is secreted in response to ROS produced by ECs and mediates ECs expression of adhesion molecule-1, activation of MMPs and inflammatory cell infiltration. Secreted CyPA activates VSMCs by ERK1/2 phosphorylation, enhances ROS production in VSMCs and promotes vascular inflammatory responses, thereby up-regulating the susceptibility of structural components of the aortic wall to aortic dissection [15]. These findings suggest that inhibition of CyPA expression in oxidative stress and inflammation-related cardiovascular disease may improve the progression of the disease.

The recruitment and transportation of leukocytes are important aspects of the inflammatory process [58]. Although chemokines are thought to be a major factor regulating cell trafficking, extracellular CyPA has recently been shown to have a direct chemical attraction to human leukocytes [59]. CyPA which is detected at high levels in tissues with ongoing inflammation, is secreted by a variety of cells [60]. EMMPRIN has been identified as the major signaling receptor for CyPA on human leukocytes. Interestingly, the expression of EMMPRIN is elevated on leukocytes from inflamed tissues, indicating a strong correlation among the presence of extracellular CyPA, EMMPRIN expression, and inflammatory responses [61]. The pro-inflammatory effect of CyPA is specifically directing chemotactic leukocytes, which can induce aggregation of neutrophils, eosinophils, T lymphocytes, and monocytes [34], activate the inflammatory signaling pathways, and promote the production of more ROS, leading to irreversible cell damage or death. CyPA promotes inflammation mainly through the following cascades: (1) CyPA binds to its receptor EMMPRIN to chemotactic inflammatory cells. Yurchenko et al. demonstrated that EMMPRIN is an essential component in the CyPA initiation signaling cascade [33]. After blocking EMMPRIN, chemotaxis of monocytes, expression of related inflammatory factors, and activation of signaling pathways induced by CyPA are inhibited [62], suggesting that the CyPA-EMMPRIN interaction is an important pro-inflammatory signal. (2) After CyPA binds to EMMPRIN, it activates the NK-κB, Akt, and MAPK signaling pathways [38,39,45] and stimulates ECs to express VCAM-1, intercellular adhesion molecule 1 (ICAM-1), and E-selectin [63]. VCAM-1 on the surface of ECs is a ligand of integrin α4β1 (VLA-4) on the surface of lymphocytes and monocytes. VCAM-1 interacts with VLA-4 and promotes the rolling and adhesion of leukocytes [64]. After inflammatory cells aggregate, they further pass through the gap of ECs and reach the subendothelium and differentiate, secreting cytokines such as tumor necrosis factor-α (TNF-α), interleukin-6 (IL-6), and interleukin-8 (IL-8), further promoting ROS production and inducing ECs activation and apoptosis [65]. (3) Activation of the CyPA/EMMPRIN pathway induces secretion of TNF-α, IL-6, and ROS, and enhances activation of MMPs [66]. ROS that come from targeted interactions with critical cysteines in the propeptide autoinhibitory domain activate MMPs post-translationally, which are normally secreted in an inactive form [67]. At the same time, inflammatory mediators, such as TNF-α and IL-6, and ROS also activate the NK-κB pathway [68]. Activation of this inflammatory signaling pathway promotes more ROS production, which forms a vicious cycle of ROS-CyPA-ROS and accelerates the progression of the inflammatory response. Activation of the inflammatory signaling pathway increases the expression of pro-hypertrophic and pro-fibrotic genes [69]. Cardiac inflammation is an important component of the pathogenesis of cardiovascular disease and an effective mediator of ROS-induced myocardial fibrosis and hypertrophy. Seizer has demonstrated that CyPA and EMMPRIN were up-regulated in patients with inflammatory cardiomyopathy and suggested that CyPA and its ligands can serve as novel diagnostic markers for oxidative stress and inflammation [70].

### CyPA promotes the proliferation of cardiac fibroblasts and has a direct hypertrophic effect on cardiomyocytes

**Figure 4:**
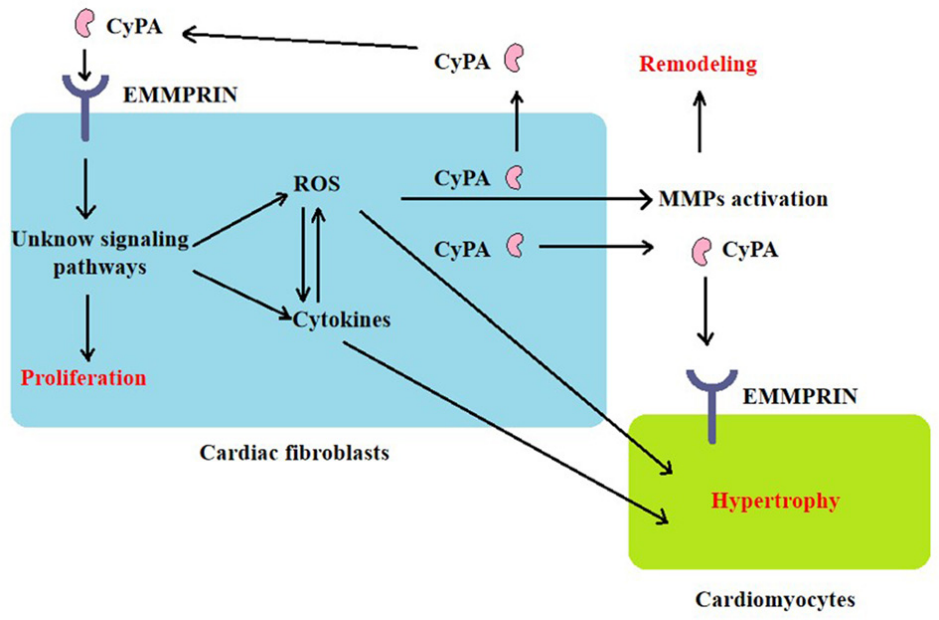
The general mechanism of CyPA-mediated cardiac fibrosis and hypertrophy CyPA promotes cardiac fibrosis activation and proliferation, which is mediated by cell surface EMMPRIN. CyPA activates certain signaling pathways and induces oxidative stress, and inflammatory cytokines, resulting in MMPs activation. ROS promotes the release of CyPA, which interacts with EMMPRIN again through autocrine mechanisms in cardiac fibroblasts and paracrine mechanisms in cardiomyocytes.

*In vitro* cell experiments have shown that culture medium prepared from AngII-stimulated cardiac fibroblasts, which were isolated from ApoE^−/−^ mice, were used to culture cardiomyocytes, and resulted in an increased myocardial hypertrophy index, such as cardiomyocyte protein synthesis and embryonic gene expression. Further experiments confirmed that cardiac fibroblasts isolated from ApoE^−/−^ mice secreted a large amount of CyPA in response to Ang II, stimulated the proliferation and migration of cardiac fibroblasts by enhancing ROS production, and the migration increased in a concentration-dependent manner. Researchers also tried to study whether cardiomyocytes secrete CyPA under the stimulation of Ang II. However, the required serum-free medium caused a large number of cardiomyocytes to die. Direct stimulation of cardiomyocytes with recombinant CyPA can lead to cardiomyocyte hypertrophy [51]. The observations above suggest that CyPA induces cardiac hypertrophy and remodeling through autocrine mechanisms in cardiac fibroblasts and paracrine mechanisms in cardiomyocytes (Figure 4), but the specific signaling pathway remains to be studied. Tian et al. confirmed that Ang II interacts with angiotensin type 2 receptor (AT2R) to stimulate ROS production in H9C2 cells and up-regulated CyPA expression [53]; Su et al. found that melatonin reduces Ang II-induced ROS production and cardiac hypertrophy in H9C2 cells by inhibiting the CyPA/EMMPRIN pathway [54]. This suggests that there may also be events in cardiomyocytes similar to the secretion of CyPA by cardiac fibroblasts, induction of oxidative stress, and cardiac hypertrophy. However, CyPA secreted by cardiomyocytes under hypoxia/reoxygenation reduced oxidative stress and apoptosis by activating the Akt pathway [71], suggesting that CyPA may play the opposite role in acute and chronic oxidative stress events. Therefore, it is necessary to study how the downstream signals of the CyPA/EMMPRIN pathway specifically regulate oxidative stress and cardiac hypertrophy, to find suitable therapeutic targets in different cardiovascular events.

ROS is essential for acetylation of CyPA and its secretion [72], while acetylated CyPA secreted extracellularly has stronger proinflammatory and oxidative stress than unmodified CyPA [73]. Inhibiting the acetylation of CyPA may improve the prognosis of patients with oxidative stress-related cardiovascular disease.

### CyPA promotes endothelial injury and vascular smooth muscle hyperplasia

In addition to cardiomyocytes and cardiac fibroblasts, mammalian myocardial tissues also include ECs and VSMCs [74]. Activation and apoptosis of vascular walls may lead to stenosis and occlusion, and further lead to excessive cardiac load, myocardial ischemia [75], cardiac hypertrophy, remodeling, and even heart failure [50].

EC dysfunction is a key event in the development and progression of vascular disease [76]. Jin et al. studied the pro-inflammatory effects of CyPA on ECs. Recombinant CyPA activated MAPK signaling pathways in cultured human umbilical vein endothelial cells, including ERK1/2, JNK and p38, and stimulated IκB-α phosphorylation and NF-κB activation. This induces the expression of adhesion molecules, including VCAM-1 and E-selectin [18]. ECs usually resist the attachment of leukocytes that flow through them. After activation, they express chemokines and adhesion molecules, leading to leukocyte recruitment [64]. The bound leukocytes migrate into the intima and monocytes (the most recruited cells) mature into macrophages, which secrete inflammatory cytokines and ROS, and activate MMPs [62]. Studies have also shown that the effect of CyPA on ECs may depend on its concentration. At low concentrations (10−100 ng/ml), CyPA increases EC proliferation, migration, invasive capacity, tubulogenesis, and the secretion of active MMP-2, which is a mediator of cell migration and angiogenesis. At high concentrations (such as 2 μg/ml), CyPA has opposite effects, reducing EC migration and viability, possibly related to the induction of Toll-like receptor-4 expression, as detected by immunocytochemistry and flow cytometry [77]. Progress in pathological cardiac hypertrophy has been considered irreversible. However, recent clinical observations and experimental studies have confirmed the reversal of pathological cardiac hypertrophy, and angiogenesis is essential in this process [6]. The pro-angiogenic effect of low concentration of CyPA in ECs suggests that it may also play a role in reversing cardiac hypertrophy and remodeling.

Abnormal VSMC growth contributes to the pathogenesis of blood vessels [75]. CyPA was first identified as a redox-sensitive medium secreted by VSMCs, activating ERK1/2, promoting DNA replication, and mediating VSMCs proliferation and hypertrophy [78]. CyPA can also promote NADPH oxidase such as p47phox translocation to the plasma membrane, produce ROS, and indirectly promote cell protein synthesis, cell growth, inflammation, and VSMC proliferation [79]. Satoh et al. found that VSMC-derived intracellular and extracellular CypA are required for the production of ROS, inflammatory cytokine expression, and activation of MMPs. Inflammation and matrix degradation in blood vessels are critical for vascular remodeling [80]. At the same time, CyPA and ROS produced by the vascular system may act on cardiomyocytes and cardiac fibroblasts, promoting the process anywhere from cardiac hypertrophy and remodeling to heart failure.

### CyPA promotes ECM dissolution

Cardiac fibroblasts are the main source of the ECM. Activated cardiac fibroblasts secrete a large amount of ECM, which promotes collagen accumulation in the myocardial interstitium (i.e, fibrosis), changes the passive stiffness properties of the myocardium, and affects ventricular compliance [81]. There are highly diverse proteolytic enzymes in the ECM, and MMPs are the most widely studied. Activated MMPs directly or indirectly proteolytically process the ECM, while certain MMP types may enhance profibrotic signaling, leading to unstable structures and abnormal functions of the ECM, and further impairing both systolic and diastolic functions [82]. There are currently about 23 types of MMPs expressed in humans, and different types of MMPs are up-regulated in different stimulation-induced cardiac hypertrophy models and exhibit diverse biological effects [83]. For example, the expression and activity of MMP-2 increase in the context of cardiac pressure overload [84], whereas in inflammatory cardiomyopathy, MMP-9 plays a key role in the poor remodeling process after myocardial injury [85]. EMMPRIN is considered to be an MMP-inducing factor involved in vascular and ventricular remodeling in cardiovascular disease. In vascular cells, cardiac fibroblasts, and monocytes, CyPA binds to EMMPRIN and induces activation of MMPs by activating signaling pathways such as the ERK1/2 and NF-κB [18,45,62,78].

The expression of CyPA and EMMPRIN in plasma of patients with acute myocardial infarction (AMI) increases. The change in CyPA concentration affects ventricular remodeling and cardiac function after AMI, which may be related to CyPA-mediated MMP-2 and MMP-9 activation [56,86]. In an animal model of AMI, intracoronary administration of CsA to inhibit CyPA reduces inflammation, down-regulates MMP expression, and reduces left ventricular remodeling [87]. The results above suggest that activation of the CyPA/EMMPRIN pathway induces secretion of inflammatory cytokines and production of ROS. The synergistic effects of inflammatory cytokines and ROS promote collagen deposition and activate MMPs, which contribute to ventricular remodeling after myocardial infarction.

Based on the up-regulation of CyPA and EMMPRIN in myocardial tissue of patients with inflammatory cardiomyopathy [70], Seizer and his team further studied the role of CyPA and EMMPRIN in mice with coxsackievirus-induced myocarditis. The results showed that compared with WT mice, inflammatory cell recruitment, MMP-9 expression, myocardial fibrosis, and contractile dysfunction were significantly decreased in Ppia^−/-^ mice, while MMP-2 expression was not significantly different between the two groups [88]. A study by Yuan showed that CyPA-EMMPRIN interaction activates MAPK/NF-κB and promotes inflammation and MMP-9 expression in monocytes [62]. MMP-9 is therefore considered to be a key proteolytic enzyme for CyPA in mediating ventricular remodeling secondary to inflammatory cardiomyopathy.

In VSMCs, CyPA binds to EMMPRIN to promote MMP-2 expression and mediate migration and proliferation of VSMCs [72]. The expression and activity of MMP-2 in plasma increase in patients with left ventricular pressure overload such as hypertension and aortic stenosis, and the level of MMP-2 is positively correlated with the development of left ventricular hypertrophy and heart failure [89,90]. While it induces local ECM degradation, MMP-2 also promotes ECM synthesis by altering intracellular signaling cascades to enhance profibrotic signaling pathways [91]. Whether CyPA promotes ventricular remodeling by up-regulating myocardial MMP-2 expression in a stress overload-induced cardiac hypertrophy model remains unclear.

## CyPA as a therapeutic target for cardiac hypertrophy and remodeling

Clinical studies have found that CyPA is significantly elevated in the plasma of patients with ventricular remodeling and heart failure caused by various diseases such as hypertension, AMI, and myocarditis [52,55,56]. A follow-up study of patients with ST-segment elevation myocardial infarction (STEMI) found that CyPA levels were positively correlated with inflammatory factors, such as IL-6 and high-sensitivity C-reactive protein (CRP), and activated MMPs. Decreased CyPA concentration at a one-month follow-up after STEMI predicted improvement of left ventricular function at 6 months [56]. CRP can only be used as an inflammatory marker to assess the level of plasma inflammation in patients with AMI. Continuously increased plasma circulating CyPA levels after STEMI may reflect a state of high oxidative stress and inflammation. ROS and inflammation further up-regulate cardiac hypertrophy and profibrotic gene expression [69]. This suggests that CyPA can be used as a marker of oxidative stress and inflammation for patients with cardiac hypertrophy and remodeling to conduct heart failure risk assessment and prognosis analysis, and provide new therapeutic targets for drugs that prevent and improve cardiac remodeling.

Early studies have shown that the Ca^2+^-calmodulin (CaM)-calcineurin (CN)-nuclear factor of activated T cell (NFAT) signaling pathway plays a crucial role in the development of cardiac hypertrophy [92]. CsA, which is an inhibitor of CN, binds to CyPA to form a dimeric complex and then binds to CN and inhibits its activity [93]. The use of CsA in animal models can effectively block or reduce cardiac hypertrophy caused by Ang II, β-AR and pressure load [94–96], but simply knocking out CN expression does not effectively improve cardiac hypertrophy [97], suggesting that CyPA may be a more important factor in promoting cardiac hypertrophy. However, CsA is immunosuppressive and therefore is not an ideal therapeutic drug [93]. NIM811, a non-immunosuppressive CsA analog, efficiently binds CyPA inside and outside the cell [98], and may bring some adverse reactions due to the inhibition of intracellular CyPA. Thus it is necessary to develop a new type of CyPA inhibitor, which is non-immunosuppressive, does not penetrate the cell membrane, and can specifically bind to extracellular CyPA (especially acetylated CyPA).

Statins such as simvastatin can inhibit oxidative stress-induced CyPA secretion in a dose-dependent manner in VSMCs likely via decreased isoprenylation of small GTPases [17]. Statins have been proven to have antioxidant effects that inhibit the development of hypertension and cardiac hypertrophy and have beneficial effects on cardiovascular changes [99]. It has been suggested that statins may play a role in improving oxidative stress and ventricular remodeling by inhibiting the secretion of CyPA.

The renin–angiotensin–aldosterone system (RAAS) plays an important role in the progression of left ventricular remodeling and heart failure after myocardial infarction. A large amount of clinical evidence indicates drugs that inhibit RAAS, such as angiotensin converting enzyme inhibitors (ACEI) and angiotensin II receptor blockers (ARB), can slow left ventricular remodeling and improve the prognosis of patients with heart failure [100]. Studies have shown that RAAS is a ROS inducer that promotes CyPA secretion [101]. Inhibition of RAAS can attenuate oxidative stress, left ventricular remodeling, and possibly reduce CyPA levels. In a rabbit model of myocardial infarction, ACEI can significantly reduce the concentration of CyPA in the left ventricular wall and improve left ventricular remodeling and left ventricular function [102].

## Conclusions and perspectives

This article describes the main mechanisms by which CyPA promotes cardiac hypertrophy and remodeling, including the promotion of oxidative stress and inflammation and the role of CyPA in the main cells (cardiomyocytes, cardiac fibroblasts, ECs, and VSMCs) in myocardial tissue, and summarizes the effects of several drugs for the treatment of heart failure on CyPA. We suggest that CyPA and its receptor EMMPRIN can be used as new therapeutic targets for preventing and improving pathological cardiac hypertrophy. However, specific signaling pathways by which the CyPA/EMMPRIN axis regulates oxidative stress and inflammation, thereby inducing cardiac hypertrophy and remodeling, are not yet known. In pathological cardiac hypertrophy caused by various factors, CyPA may activate different types of MMPs to mediate myocardial fibrosis and remodeling. At present, most of the viewpoints suggest that CyPA plays a role of promoting inflammation and oxidative stress in the development of pathological cardiac hypertrophy, but the pro-angiogenic effect of low concentration of CyPA in ECs suggests that it may have an advantage in promoting the regression of pathological cardiac hypertrophy. Further basic science and clinical trials are required to elucidate the specific mechanisms by which CyPA/EMMPRIN promotes pathological cardiac hypertrophy under different pathophysiological conditions, which will be of great significance for the development of new treatments that prevent or reverse cardiac remodeling.

## Abbreviations

ACEI: angiotensin converting enzyme inhibitor
ARB: angiotensin II receptor blocker
CyPA: Cyclophilin A
CyP: cyclophilin
ECM: extracellular matrix
MMP: matrix metalloproteinase
RAAS: renin–angiotensin–aldosterone system
ROS: reactive oxygen species
